# Assessing the Readability of Covid-19 Testing Messages on the Internet

**DOI:** 10.1007/s10900-021-00973-6

**Published:** 2021-02-27

**Authors:** Philip Garcia, Joseph Fera, Jan Mohlman, Corey H. Basch

**Affiliations:** 1 Department of Public Health, Icahn School of Medicine at Mount Sinai, 17 E 102nd Street 5th Floor West, New York, NY 10029 USA; 2 Department of Mathematics, Lehman College, The City University of New York, Bronx, NY 10468 USA; 3 Department of Psychology, William Paterson University, Wayne, NJ 07470 USA; 4 Department of Public Health, William Paterson University, Wayne, NJ 07470 USA

**Keywords:** COVID-19 testing, Readability, Internet, Online

## Abstract

The COVID-19 pandemic first became evident at the end of 2019, and because of the many unknown aspects of this emerging infectious disease, the internet quickly became a source of information for consumers. It is important for any vital information to be written unambiguously, and at a level that can be understood by all people regardless of education levels. The purpose of this study was to assess the readability of 50 sources of COVID19 testing information online. Only 6 websites out of 50 received an appropriate readability score on more than one assessment. One-sample, one-tailed t-tests (α = 0.05, df = 49) were used to see if the websites with information on COVID-19 testing are being written at appropriate reading levels. The resulting *p*-values indicate that each *p*-value recorded is substantially below 0.05, it is very unlikely that websites on this topic are being written at the recommended levels. Even the optimal messages on COVID-19 reflect a confusing and rapidly changing public health crisis, however if messages are kept simple and clear, individuals will have the best possible chance of optimizing behavioral mitigation strategies. These are compelling reasons for informational hosts to take necessary steps to ensure that messages are written in as simple terms as possible. To this end, it is suggested that internet sites dispersing COVID-19 testing information build in text analysis methods for all published messages, particularly those meant to inform best health practices in the time of a pandemic.

## Introduction

The first announcements of an illness causing pneumonia-like symptoms had emerged in Wuhan China were evident at the end of 2019 [1, 2] and the illness was later identified as COVID-19, caused by the SARS-CoV-2 virus. Now a pandemic, [3] with 90,343,519 confirmed cases of COVID-19 and 1,936,133 deaths worldwide as of January 11, 2021 [4]. In the United States, specifically there have been 22,409,480 confirmed cases of COVID-19 and 374,341 deaths [4]. The need for adequate testing measures quickly became clear, and on March 21, 2020, the World Health Organization (WHO) highlighted that there were shortfalls in testing capacity [1]. Their laboratory testing strategies were expanded upon and reiterated as the pandemic persisted [1].

According to the Food and Drug administration, at the current time in the United states, “there are two types of diagnostic tests – molecular tests, such as RT-PCR tests, that detect the virus’s genetic material, and antigen tests that detect specific proteins from the virus [5].” Additionally, although not diagnostic, antibody tests determine if a person has developed antibodies indicating a person has had the virus. Molecular tests such as the reverse transcription molecular polymerase chain reaction (RT-PCR) and antigen tests indicate if a person is currently infected and antibody testing discerns if a person has had an infection in the past [5]. Most molecular PCR and antigen tests utilize a nasal swab to gather respiratory fluid and cell samples, whereas antibody tests use a blood draw or finger stick [5].

Despite the development of tests for COVID-19, the testing process has not been so straightforward. Challenges include, but are not limited to timing of the sample [6, 7] cost, availability, and placement of testing location centers [8], personnel issues [9, 10], variation in sensitivity and specificity [11, 12], and lag time for results [13]. Given these challenges and changes to guidelines for testing [14], the general public was left seeking answers to questions related to testing. An increasing number of people turn to the internet for information. In fact, 35 % of U.S. adults indicate that they have used the internet as a source of medical information, often without verifying their findings with a physician [15] despite the fact that this information guides medical decisions [16].

Often, medical information is difficult to understand, and even though it is highly accessible to a lay audience, few U.S. adults have proficient levels of health literacy [17]. Often health information on the internet is written at levels that are difficult to read for the average adult. The importance of COVID-19 testing in reducing community transmission and saving lives cannot be understated. [18] Therefore, it is important that online information on this topic be as accessible as possible. Therefore, the purpose of this study was to determine the level of difficulty of written information about COVID testing that is readily available on the internet.

## Methods

The methods for this study follow our prior work on general COVID-19 information [19]. Fifty English language websites were gathered by the researcher to establish the sample size for the study. Google.com was utilized as the preferred search engine, primarily due to its popularity and accessibility across all populations. The search keywords used to generate the sample size was “COVID 19 testing.” URLs of the first eligible 50 websites were recorded in a Microsoft Excel spreadsheet. Sponsored or advertised weblinks were excluded from the sample. Additionally, those webpages functioning as home pages for other COVID 19 testing related resources were also excluded. For a website to qualify and be eligible in the study sample, the page at minimum, must have any direct information regarding COVID 19 testing. Overall, a total of 8 search result pages were reviewed, with 15 skipped websites that met the exclusionary criteria of a home page. Of note, these 15 websites do not include any sponsored or advertised URLs as they were not tracked for this study.

The preferred browser utilized in this study was Google Chrome, attributed by its reliability, user friendliness, speed, and security. Search results have the potential to be uniquely tailored to the user according to any preferences, saved data, and search histories. To prevent this, prior to conducting the search, Google Chrome was first cleared of all browsing and search history, cache, cookies, and other data.

Readable.io [20] was used as the readability scoring tool. The Readable service generates five common and recommended readability tests: Flesch-Kincaid Grade Level (FKGL), Gunning Fog Index (GFI), Coleman-Liau Index (CLI), the Simple Measure of Gobbledygook (SMOG) Grade level, and Flesch-Kincaid Reading Ease (FRE). FKGL and FRE are identical in that they are intended to calculate and analyze sentence word length, but differ in their scoring schema. FKGL develops a score similar to a United States grade level, while FRE deploys a ranged scoring system (0-100) where higher results indicate a generally easier read, fit for the average consumer, whereas lower scores indicate content best suited for university graduates or professionals. GFI similarly deploys a grade level scoring pattern with a focus on measuring the average words per sentence in addition to the number of polysyllabic words. CLI calculates the number of letters in a word and number of words in a sentence, which is then scored through the use of a grade level pattern similar to the United States Education system [20]. Lastly, the SMOG grade level uses a syllable counting system with a subsection of text to establish the lowest education level to understand the material [21]. These five readability scores were calculated for each of the 50 websites.

Microsoft Excel was utilized for all data capture tasks. The researcher created a code book with each row capturing different categories. These categories include: website characteristics like type (.gov, .com, .edu, etc.), creation/written dates, as well as the results of the readability tests (FKGL, GFI, CLI, SMOG, and FRE). As per the protocol at William Paterson University, the Institutional Review Board does not review studies that do not involve human subjects.

## Results

In total, there were 50 websites on COVID-19 testing included in this readability study. For each site, a readability score was determined using five different readability assessments: FRE, FKGL, GFI, CLI, and SMOG. The FRE test is scored from 0 to 100, with scores from 80 to 100 considered to be easy-to-read, 60–79 average-to-read, and 0–59 difficult-to-read. The remaining four measures assign a score based on reading grade level, i.e. 7 indicates a grade 7 reading level. Based on recommendations from public health professionals, the analysis here considers a 6th grade reading level or an FRE score of easy to be appropriate [22]. All data entry and analysis was completing using Microsoft Excel.

Overall, the analysis indicates that online materials for COVID-19 testing are not being written at an appropriate reading level. None of the websites reviewed tested at the recommended level on all five readability assessments. In fact, only 6 websites received an appropriate readability score on more than one assessment. Of these 6, two were deemed appropriate by 3 tests (FKGL, GFI, and CLI) and four were deemed appropriate by 2 tests (FHGL and GFI). The remaining 9 received an appropriate score by only one of the five assessments.

Table 1 displays the distribution of websites by reading assessment and subsequently by difficulty level. The FRE, CLI, and SMOG characterized a majority of the sites as difficult to read. The FKGL, on the other hand, classified a majority of the sites (54 %) as average and falling within the 7th to 10th grade reading level range.

**Table 1:** The distribution of websites by readability assessment and then by difficulty level

Readability	Count (n = 50)	%
*Flesch-Kincaid Reading Ease*		
Easy (80–100)	0	0 %
Average (60–79)	16	32 %
Difficult (0–59)	34	68 %
*Flesch-Kincaid Grade Level*		
Up to grade 7	11	22 %
Grades 7–10	27	54 %
Beyond grade 10	12	24 %
*Gunning Fog Index*		
Up to grade 7	10	20 %
Grades 7–10	21	42 %
Beyond grade 10	19	38 %
*Coleman-Liau Index*		
Up to grade 7	2	4 %
Grades 7–10	16	32 %
Beyond grade 10	32	64 %
*Simple Measure of Gobbledygook*		
Up to grade 7	0	0 %
Grades 7–10	17	34 %
Beyond grade 10	33	66 %

One-sample, one-tailed t-tests (α = 0.05, df = 49) were used to see if the websites with information on COVID-19 testing are being written at appropriate reading levels. The resulting *p*-values are included in Table 2 along with mean readability score and standard deviation information. Given that each *p*-value recorded is substantially below 0.05, it is very unlikely that websites on this topic are being written at the recommended levels.

**Table 2:** Mean and Standard Deviation Statistics

Readability Test	Mean	Standard Deviation	*p-value*
Flesch-Kincaid Grade Level	8.62	2.0753	1.87345E-07
Gunning Fog Index	9.42	2.7342	1.82437E-08
Coleman-Liau Index	10.70	2.0211	3.6206E-18
Simple Measure of Gobbledygook	11.09	1.7965	6.02558E-22
Flesch-Kincaid Reading Ease	54.35	10.2355	2.97216E-23

Table 3 shows the distribution of the 50 websites by URL extension. Roughly half of sites (n = 24) had a .com extension and roughly half (n = 26) had an extension other than .com. These included .org. .gov, .net, .edu, and other. To determine if there was any difference in readability score between .com sites and non-.com sites, one-sided two-sample t-tests (α = 0.05) were performed for each readability test. The resulting *p*-values and additional descriptive statistics are given in Table 4. Note that a significant difference was only found for one readability test: the GFI (*p* = .0296). Thus, it appears that .com sites on COVID-19 do have significantly higher GFI scores than non-.com sites on the same material. Still, both categories had average GFI scores above what is considered appropriate.

**Table 3:** The distribution of the sites sampled by URL extension

Website Type	Count	%
.com	24	48 %
.org	9	18 %
.gov	13	26 %
.net	1	2 %
.edu	1	2 %
other	2	4 %

**Table 4:** Readability for .com vs. non-.com sites

Readability	.com		Other		p-value
Test	Mean	St. Dev	Mean	St. Dev	
Flesch-Kincaid Grade Level	9.04	1.9013	8.23	2.1137	0.0860
Gunning Fog Index	10.18	2.4821	8.72	2.7175	0.0296
Coleman-Liau Index	10.97	1.3840	10.45	2.4087	0.1815
Simple Measure of Gobbledygook	11.49	1.6435	10.73	1.8197	0.0681
Flesch-Kincaid Reading Ease	54.12	8.1476	54.55	11.6652	0.4407

## Discussion

Results of this analysis showed that the COVID-19 testing information reviewed on 50 prominent internet sites was written at reading levels considerably higher than recommended. This represents a miscalculation of either the text composition or readership of messages on the part of the hosts; either of which poses an obstacle to the mitigation of illness through behavioral strategies such as testing. It is known that simple, well composed messages can facilitate the management of public health crises. However, because information on COVID-19 is inherently protean and politicized, conflicting messages and contingencies have left many readers paralyzed in terms of best health practices [23]. Dissemination of complicated messages about testing thus add to the already stressful and confusing informational landscape of COVID-19. These problems with internet-based messaging must be corrected as soon as possible, particularly due to the forthcoming need to control vaccine distribution across the U.S. through similar communications.

In addition to being written at a more difficult reading level than advised, it is likely that other obstacles to comprehensible coronavirus testing messages are in effect. For instance, it is known that emotional states such as anxiety motivate individuals to seek out information on novel or ambiguous sources of threat [24–26]. At the same time, however, anxiety and stress can interfere with the visual processing and interpretation of messages related to threat [27, 28]. This can create a powerful feed-forward loop of COVID-19 information seeking, detection of complicated information on internet sites, even more of a rise in anxiety levels, and the cycle repeats, ultimately resulting in a failure of informed health behaviors related to the ongoing pandemic [19]. Objective evidence of the difficulty of understanding COVID-19 messages is found in the development of special tools meant to assist readers in deciding whether or not to obtain a test [29].

Even the optimal messages on COVID-19 reflect a confusing and rapidly changing public health crisis [30], however if messages are kept simple and clear, individuals will have the best possible chance of optimizing behavioral mitigation strategies. These are compelling reasons for informational hosts to take necessary steps to ensure that messages are written in as simple terms as possible [31]. To this end, it is suggest that internet sites dispersing COVID-19 testing information build in text analysis methods for all published messages, particularly those meant to inform best health practices in the time of a pandemic.
